# Colonization prevalence and antibiotic susceptibility of Group B *Streptococcus* in pregnant women over a 6-year period in Dongguan, China

**DOI:** 10.1371/journal.pone.0183083

**Published:** 2017-08-15

**Authors:** Wenjing Ji, Lihua Zhang, Zhusheng Guo, Shujin Xie, Weiqing Yang, Junjian Chen, Jiamin Wang, Zhiqin Cheng, Xin Wang, Xuehai Zhu, Jianwen Wang, Haiqing Wang, Juan Huang, Ning Liang, David J. McIver

**Affiliations:** 1 Department of Pharmacy Administration and Clinical Pharmacy, School of Pharmacy, Xi’an Jiaotong University, Xi’an, China; 2 Center for Drug Safety and Policy Research, Xi’an Jiaotong University, Xi’an, China; 3 Department of Clinical Laboratory, Donghua Hospital, Dongguan, China; 4 Department of Microbiology, Guangdong Medical College, Dongguan, China; 5 Metabiota Inc., Nanaimo, Canada; Universidad Nacional de la Plata, ARGENTINA

## Abstract

This study investigated the prevalence of recto-vaginal Group B *Streptococcus* (GBS) colonization, serotype distribution, and antimicrobial susceptibility patterns among pregnant women in Dongguan, China. Recto-vaginal swabs were collected from pregnant women at gestational age 35–37 weeks between January 1^st^ 2009 and December 31^st^ 2014. Isolates were serotyped by latex-agglutination and were tested against seven antimicrobials by disk diffusion. Of 7,726 pregnant women who completed GBS testing, 636 (8.2%) were GBS carriers. Of 153 GBS isolates available for typing, 6 serotypes (Ia, Ib, III, V, VI and VIII) were identified with type III being predominant, while 9 (5.9%) were non-typable isolates. All isolates were sensitive to penicillin, ceftriaxone, linezolid and vancomycin, whereas 52.4% were resistant to clindamycin, 25.9% were resistant to levofloxacin and 64.9% were resistant to erythromycin. This study showed the recto-vaginal colonization prevalence of GBS in Dongguan is significant. Due to 100% susceptibility to penicillin of all GBS samples, penicillin remains the first recommendation for treatment and prevention against GBS infection. Susceptibility testing should be performed for women allergic to penicillin in order to choose the most appropriate antibacterial agents for treatment and prevention of vertical transmission to neonates. In addition, we suggest establishing standard processes for GBS culture and identification in China as early as possible.

## Introduction

Group B *Streptococcus* (GBS) bacteria cause invasive disease primarily in infants, pregnant or postpartum women, and older adults, with the highest incidence in the United States being among black infants [1]. Maternal colonization with GBS in the genitourinary or gastrointestinal tract is the primary risk factor for the disease, and it is generally accepted that the major route of newborns acquiring early onset GBS disease is vertical transmission from colonized mothers [2–4]. Epidemiological studies have revealed that pregnant women colonized with GBS are 25-times more likely to deliver infants with early onset of GBS disease (EOD, occurring before 7 days of age) than women with negative prenatal cultures [5, 6]. An estimated 20–30% of pregnant women in developed countries are colonized with GBS and approximately 50% of their babies become colonized during delivery [7, 8]. The introduction of routine screening for rectovaginal colonization in late pregnancy (35–37 weeks) and intrapartum antibiotic prophylaxis (IAP) administration at delivery has significantly reduced the incidence of EOD in countries where it has been implemented [1, 9, 10]. However, there is a lack of understanding of GBS epidemiology characteristics in China, and limited data is available. At present, there are no specific guidelines for GBS screening and prevention in China, and no surveillance program exists to monitor the prevalence of GBS infection among pregnant women or infants [11]. It is essential to understand the colonization prevalence of pregnant women in order to prevent adverse outcomes in pregnant women and infants. Equally, research is needed to understand local patterns of antibiotic resistance to choose appropriate treatments. This study aimed to investigate the prevalence of colonization with GBS of pregnant women in Dongguan and identify GBS serotypes, to provide evidence for the development of strategies to produce interventions. In addition, we explored GBS susceptibility to seven common antibiotics to guide intrapartum antibiotic prophylaxis, and ultimately to reduce newborn EOD infection.

## Materials and methods

### Study design and ethical approval

We retrospectively collected data on pregnant women who had received a prenatal examination at Donghua Hospital between January 1^st^ 2009 and December 31^st^ 2014. Donghua hospital is located in Dongguan, Guangdong province of China, which is a major city in southern mainland China. The hospital has 120 beds in the obstetrical department, with nearly 4,000 mothers delivering annually, and the hospital catchment area supports a population of around 300,000 people in Dongguan. The Donghua hospital, as a large urban general hospital with advanced medical facilities, is also an affiliated hospital of Sun Yat-Sen University.

GBS testing was implemented among pregnant women at 35–37 weeks gestation in Donghua Hospital, according to the recommendation from the United States Centers for Disease Control and Prevention’s revised guidelines for the prevention of early-onset GBS disease [9]. If the result of GBS screening was positive, the doctor offered intrapartum antibiotic prophylaxis for the GBS colonized women. Penicillin was the first choice to be used for three days to reduce the incidence of neonatal EOD. This study was approved by the Ethics Committee of Donghua Hospital on March 26th, 2016. We collected data and finished sample serotyping from April to August in 2016.

### Sample collection and GBS isolate culture

A physician collected a rectovaginal swab for GBS culture by initially swabbing the vaginal introitus and thereafter the rectum (through the anal sphincter). Swabs were placed in 2ml of processing solution containing 0.16 mg/ml gentamicin saline for one minute and incubated at 35°C in 5–10% CO_2_ for 18–24 hours. A solution with a ratio of 20:1 of gentamicin saline to medium was created, the specimen was washed for 1 minute, and then the bacterial culture was added. The effect of washing the sample with gentamicin was tested by a mixed bacteria solution composed of standard strains of *Staphylococcus aureus*, *Escherichia coli*, *Streptococcus pneumoniae*, *Enterococcus faecalis* and GBS. This validated method inhibited the growth of non-GBS bacteria and allowed GBS to grow. (Refer to S1 File)

Broths were subcultured onto Columbia blood agar, and isolates were identified based on colony morphology and β-hemolysis. We used the CAMP test to separate suspicious strains with a thin line method of inoculation to the blood on the plate, and then produced *β-hemolysin staphylococcus* inoculation of a line, which is perpendicular to the first sample line but does not contact it. Cultures were incubated at 37°C for 12 hours. Two strains of bacterial growth line separation of the region showed significant hemolysis was present, which proved to be for the GBS. If the test result was negative, then re-identification was performed by using a BioMerieux VITEK32 (BioMerieux, France).

Due to limited funding, only a subset of strains from 2013 was selected for serotyping. Of the isolates available from 2013, we selected the first 23 cases of each quarter as the samples to serotype, in order to achieve a representative sample, while all GBS isolates identified in 2014 were serotyped. Serotypes were classified as Ia, Ib, II, III, IV, V, VI, VII, VIII, IX, or Nontypable (NT), using the rapid latex agglutination test (Strep-B-Latex kit; Statens SerumInstitut, Copenhagen, Denmark) according to the manufacturer’s instructions.

### Antimicrobial susceptibility testing

Isolates were tested against penicillin, ceftriaxone, levofloxacin, clindamycin, erythromycin, linezolid, and vancomycin, by the K-B disk diffusion (Oxoid Limited, United Kingdom) method according to Clinical and Laboratory Standards Institute (CLSI) 2009 guidelines, using *Streptococcus pneumoniae* ATCC49619 as a control strain[12]. The criteria for GBS antimicrobial sensitivity patterns are listed in the Table 1. WHONET 5.6 was used for statistical analysis.

**Table 1:** Criteria for GBS antimicrobial sensitivity patterns.

Antimicrobial	Drug concentration(μg)	Diameter of the inhibition zone (mm)		
		Sensitive	Intermediate	Resistant
**Penicillin**	10	≥24	-	-
**Ceftriaxon**	30	≥24	-	-
**Levofloxacin**	5	≥17	14–16	≤13
**Clindamycin**	2	≥19	16–18	≤15
**Erythromycin**	15	≥21	16–20	≤15
**Linezolid**	30	≥21	16–20	≤15
**Vancomycin**	30	≥17	-	-

### Statistical analysis

The prevalence of maternal GBS colonization was calculated as the number of pregnant women, at 35–37 weeks of gestation, who were GBS positive out of the women screened at Donghua hospital over the 6-year study period. Serotype distribution is described as a frequency and percentage, and Chi-square or Fisher’s exact test were used to compare serotype distribution between years. The Cochran-Armitage trend test was used to evaluate annual trends of antibiotic susceptibility. All analyses were completed with SAS 9.0, and two-tailed *P*<0.05 were considered statistically significant.

## Results

### Colonization prevalence

A total of 27,454 women delivered at Donghua hospital over the 6-year period. Among these, 7,726 (28.1%) pregnant women at 35–37 weeks of gestation, aged 16–47 years (28.5±4.4), completed GBS testing. 636 (8.2%) of the women were GBS positive, with the highest prevalence observed in 2009 (11.4%) and the lowest in 2010 (6.3%) (Table 2). The main reason for the small sample size in 2009 (746) was that this was the first year of implementation of GBS screening in our hospital, and the sensitization to pregnant women was not optimized. Similarly, a small sample size in 2014 (733) was due to the Dongguan Women and Children’s Hospital being relocated near to the Donghua hospital, which lead to a decrease in the number of pregnant women attending Donghua hospital. When excluding data from the first and last years, to examine the impact these low-enrollment periods had on overall results, the resulting GBS colonization prevalence was 7.8% (CI 7.18–8.51), which was not statistically significantly different compared to the overall prevalence (8.2%, CI 7.61–8.85, *P* = 0.402).

**Table 2:** Prevalence of GBS colonization of perinatal pregnant women from 2009 to 2014.

Year	# Sampled	# Cultured Positive	Colonization Prevalence% (95% CI)
**2009**	746	85	11.4 (9.11–13.67)
**2010**	1,663	104	6.3 (5.09–7.41)
**2011**	1,353	108	8.0 (6.54–9.42)
**2012**	1,884	148	7.9 (6.64–9.08)
**2013**	1,347	130	9.7 (8.07–11.23)
**2014**	733	61	8.3 (6.33–10.32)
**Total**	7,726	636	8.2 (7.61~8.85)

### Serotype distribution

A total of 153 GBS isolates were serotyped in the present study; 92 strains from 2013 (23 from each quarter), and 61 from 2014. Serotypes Ia, Ib, III, V, VI, and VIII were identified, while 9 (5.9%) were non-typable isolates. More than half, 54.9%, were serotype III (84/153), 17.7% were serotype Ia (27/153), 13.1% were serotype Ib (20/153), 6.5% were serotype V (10/153), 1.3% were serotype VI (2/153), and 0.7% were serotype VIII (1/153). The distribution of GBS serotypes showed no statistically significant difference between 2013 and 2014 (*P* = 0.997) (Table 3).

**Table 3:** Serotype distribution of GBS strains from 2013 and 2014.

Serotype	2013 (n = 92)	2014 (n = 61)	Total (n = 153)
	n (%)	n (%)	n (%)
**Ia**	15 (16.3)	12 (19.7)	27 (17.6)
**Ib**	12 (13.0)	8 (13.1)	20 (13.1)
**III**	51 (55.4)	33 (54.1)	84 (54.9)
**V**	6 (6.5)	4 (6.6)	10 (6.5)
**VI**	1 (1.1)	1 (1.6)	2 (1.3)
**VIII**	1 (1.1)	0 (0.0)	1 (0.7)
**NT**	6 (6.5)	3 (4.9)	9 (5.9)

We compared the differences in the serotype pattern across the quarters between 2013 and 2014, to determine whether the GBS serotype varied by season or quarter of the same year, and no significant differences were observed.

### Antimicrobial susceptibility

**Fig 1:**
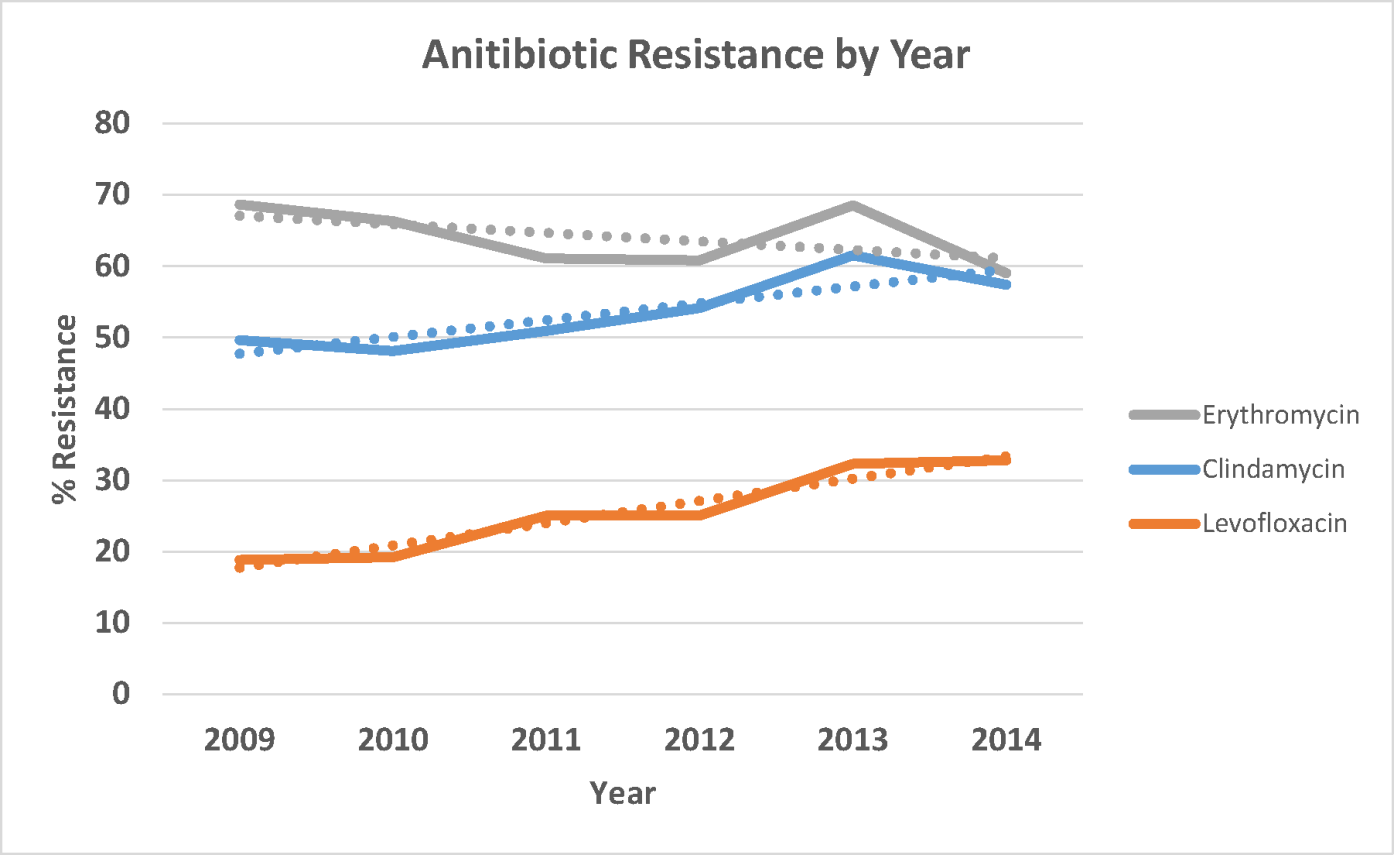
Prevalence of GBS antibiotic resistance (with linear trend lines), by year, of 636 isolates. The proportion GBS isolates resistant to erythromycin decreased between 2009 and 2014 (z = 0.665, *P* = 0.506), while an increase in clindamycin and levofloxacin resistance was observed over the same period (z = 2.097, *P* = 0.036, and z = 2.857, *P* = 0.004, respectively).

All 636 identified GBS isolates were tested against penicillin, ceftriaxon, levofloxacin, clindamycin, erythromycin, linezolid, and vancomycin. All isolates were sensitive to penicillin, ceftriaxone, linezolid, and vancomycin; 64.9% were resistant to erythromycin, 52.4% to clindamycin, and 25.9% were resistant to levofloxacin. There was no statistically significant difference in erythromycin resistance between 2009 and 2014 (z = 0.665, *P* = 0.506), while resistance to clindamycin and levofloxacin significantly increased over the same period (z = 2.097, *P* = 0.036, and z = 2.857, *P* = 0.004, respectively) (Table 4 and Fig 1).

**Table 4:** GBS antimicrobial sensitivity patterns.

Antimicrobial	Sensitive, n (%)	Intermediate, n (%)	Resistant, n (%)
**Penicillin**	636 (100)	0	0
**Ceftriaxon**	636 (100)	0	0
**Levofloxacin**	461 (72.5)	10 (1.6)	165 (25.9)
**Clindamycin**	274 (43.1)	29 (4.6)	333 (52.4)
**Erythromycin**	150 (23.6)	73 (11.5)	413 (64.9)
**Linezolid**	636 (100)	0	0
**Vancomycin**	636 (100)	0	0

## Discussion

GBS colonization in pregnant women is the primary risk factor for EOD in infants [5]. A review by Stoll reported that overall maternal GBS colonization rate in developing countries was 12.7% [13]. Barcaite reported that the colonization rate in European countries ranged from 6.5% to 36.0% [14]. A more recent systematic review and meta-analysis by Kwatra et al. found the estimated mean prevalence of rectovaginal GBS colonization was 17.9% overall and was highest in Africa (22.4%) and lowest in southeast Asia (11.1%) [15]. Although there are some studies reporting on maternal colonization in perinatal pregnant women in China [16], data is still very limited and more research is needed.

In this study, the average colonization prevalence was found to be 8.2%, which is lower than the estimated mean prevalence globally and within southeast Asia [15], but similar to other Asian countries such as Korea (8.0%) [17], Japan (8.2%) [18], Myanmar (7.1%) [19], and Philippines (7.5%) [19], and also close to that of Beijing’s prevalence of 6.5–11.1% [16, 20, 21]. Meanwhile, the observed GBS prevalence in this study is lower than in Dongying of Shandong province, China (32.2%) [22]. These results indicate that the reported GBS colonization prevalence in China varies greatly between regions. Besides the possible influence of colonization prevalence caused by regional differences, other factors may also affect prevalence distribution (e.g. sampling period, collection site of specimens, collection and culture method for GBS isolation and identification, etc.). Lu’s study [16] has the same targeted population and same method for sample collection and culture as this study, and GBS carrier prevalence were similar (7.1% and 8.2%). However, studies by Ma [20] and Fu [22] only collected vaginal samples rather than vaginal-rectal swabs. Antibiotic usage before sample collection is another potential influencing factor; however, only Fu’s study described that their study population did not use any antibiotics within two weeks before sample collection. Besides these, many other factors contribute to different colonization rates during pregnancy, such as maternal age, marital status, frequency of intercourse with multiple partners, and others [22]. Currently in China, an important issue is that the National Clinical Laboratory Operation Practice does not specify GBS culture methods, resulting in non-standardized analyses in China. To our knowledge, only vaginal swabs, rather than rectovaginal swabs like this study, are collected in most Chinese hospitals, which probably contribute to the varying GBS colonization prevalence between regions and countries.

The present study performed serotyping for 153 GBS isolates collected in 2013–2014, and identified 6 serotypes with serotype III accounting for 54.9% of isolates overall. The proportion of three serotypes (Ia, Ib, III), which accounted for 85.6% of the total, is higher than reported in Beijing (accounting for 75.1% of all serotypes) [16]. Lu’s study in Beijing also found that serotypes II (14, 7.0%), IV (1, 0.5%), and NT (1, 0.5%) strains made up 8.0% of 201 strains [16]. The observed proportion of serotypes Ia, Ib and III is also higher than South Africa (75.7%) [23], Canada (54.1%) [24], Germany (63.2%) [25], the United Kingdom (67.9%) [8], Japan (49.4%) [26] and South Korea (69.2%) [17]. Our study did not identify serotypes II and IV, but NT accounted for 5.9% of isolates. Non-typable strains of GBS have been observed in other studies as well [16, 27, 28], and our finding of 5.9% NT isolates falls within the range reported from other studies and countries, ranging from 13.9% in Canada to 5.6% in Asia [27]. GBS isolates that are NT due to the presence of mutations or insertion sequences in the cps genes have also been described previously [3, 29]. In general, serotypes Ia, Ib, and III are predominant and serotypes VI, VII, and VIII are relatively rare. Serotype II isolates in the Americas and Europe are higher than other regions [15].

It has been reported that none of the mothers of infants who developed GBS disease had antibodies to type III capsular polysaccharide (CPS) [30]. Hence, vaccination of pregnant women against GBS might be an effective method to protect neonates and their mothers against infection. An appropriate vaccine development has reached a randomized phase 1b/2 trial [31]. The potential trivalent (Ia, Ib, III) GBS vaccine would cover more than 85% of observed serotypes in Dongguan. Based on our finding that the general serotype distribution across quarters and years had not changed much between 2013 and 2014, the serotyping in this study provides a reference value in Dongguan.

In the present study, 100% of isolates were sensitive to penicillin, ceftriaxone, linezolid and vancomycin. Both penicillin and ampicillin were effective for treatment and prevention of GBS as the first-line treatment in pregnant women and neonates [9]. Penicillin has a narrow spectrum of antimicrobial activity and therefore might be less likely to select for resistant organisms compared to other options. For penicillin-allergic women, cefazolin, clindamycin and erythromycin can be useful alternatives. However, the proportions of GBS isolates with resistance to erythromycin, clindamycin or levofloxacin have increased over the past 20 years [4] In the present study, 64.9% were resistant to erythromycin, 52.4% to clindamycin, and 25.9% to levofloxacin, and the resistance to clindamycin and levofloxacin increased significantly over the six years of observation. Therefore, addressing GBS infection should be based upon the sensitivity tests of antibiotics. In general, penicillin is still the first recommendation for treatment and prevention against GBS infection in Dongguan. Erythromycin and clindamycin susceptibility testing should be performed for women allergic to penicillin, to choose the most appropriate antibacterial agents for treatment and prevention against GBS infection.

GBS screening performed among 7,726 pregnant women in the study hospital is helpful to understand the epidemiology of GBS colonization in Donghua. Screening results supported the implementation of IAP to prevent adverse outcomes in both screened pregnant women and infants, and in the long run, GBS screening results would provide evidence for developing guidelines for GBS surveillance and prevention in China.

### Strengths and limitations

GBS disease has not been well characterized in China and this is the first long-period study to explore the GBS colonization prevalence and antimicrobial resistance patterns among pregnant women in Dongguan, China. The findings provided justification for taking preventive measures for GBS disease. The results of antimicrobial resistance showed the local GBS resistance patterns, which we anticipate will promote the rational use of antibiotics in Dongguan. There are also several limitations to this study. Firstly, selection bias is an inevitable limitation, as the subjects in this study at Donghua Hospital, which is an urban tertiary hospital from southern China, will be biased towards women in urban China. Furthermore, only 28.1% of pregnant women admitted to Donghua hospital took the GBS test over the 6-year period. One of main reasons was that pregnant Chinese women have a lack of awareness about the severity of GBS disease and the importance of its prevention, which influenced them to believe that GBS screening was not necessary. On the other hand, patients also need to pay for the GBS screening fee by themselves because health insurance does not cover the GBS test. Secondly, due to only 153 (22.5%) of 636 strains being serotyped in this study, caution should be used when extrapolating these findings to the population level. Thirdly, we did not collect data on GBS infections among newborns of the screened and non-screened mothers in this study. Our research team is planning to conduct a prospective case-control study to fill the research gap in the near future.

## Supporting information

S1 File GBS culture method. (DOCX)

S1 Database GBS colonization. (XLS)
