# Supplementary material for: Colonization prevalence and antibiotic susceptibility of Group B Streptococcus in pregnant women over a 6-year period in Dongguan, China
Source: PLoS One. 2017 Aug 15;12(8):e0183083. doi: 10.1371/journal.pone.0183083 (PMC5557540; doi:10.1371/journal.pone.0183083)
Supplement: S1 File — (DOCX) [file pone.0183083.s001.docx]

The detailed experimental procedure is as follows:

1. The standard strains of *Staphylococcus aureus, Escherichia coli, Streptococcus pneumoniae, Enterococcus faecalis* and Group B *Streptococcus* were divided into 0.5 Maxwell units, the bacteria were taken 1ML respectively and fully mixed, then corrected to 0.5 Maxwell units. At this time, the solution contains about 1.5*10^8^CFU/ml of bacteria. Remove 0.1 ml of bacteria into 9.9 ml sterile saline, shake, and the resulting solution contains about 1.5*10^6^CFU / ml. Remove 0.1 ml of solution into 9.9ml sterile saline, shake, and the resulting solution contains about 1.5*10^4^CFU/ml of bacteria. Take 2 ml of solution into 2 ml of sterile saline, and then the solution contains about 7500CFU / ml of bacteria. The other six concentrations were prepared in a similar method, and sterile sampling swabs were placed in each concentration tube.
2. Preparation of Gentamicin containing saline: one 2 ml of 80,000 units of gentamicin sulfate injection was added to 500 ml of physiological saline (equivalent to 1 ml of saline containing 0.16 mg of gentamicin sulfate), each for 2ml as a reserve.
3. A total of 7 concentrations from high to low were then divided into two groups (group A and group B), Group A was a non-washing group: the sample swabs in each concentration of bacteria were placed in TH enrichment solution for enrichment. In group B, the sample swabs in each concentration of bacteria were placed in 2ml saline containing gentamicin for 1 minute, then placed in TH enrichment solution for enrichment, incubated at 35℃ for 18 - 24 hours, then 10μl were transferred to Colombian blood plate and cultured at 35℃ for 18 - 24h, observe whether GBS colonies were grown (Table 1).

Table 1 The growth and separation of GBS mixed with equal amount of bacteria in TH enrichment solution

| **Group** | **Concentration (Cfu/ml)** | | | | | | |
| --- | --- | --- | --- | --- | --- | --- | --- |
|  | **7500** | **3750** | **1875** | **937.5** | **468.8** | **234.4** | **117.2** |
| Group A (Non-washing) | Non-GBS bacterial growth | Non-GBS bacterial growth | Non-GBS bacterial growth | Non-GBS bacterial growth | Non-GBS bacterial growth | Non-GBS bacterial growth | Non-GBS bacterial growth |
| Group B  (Washing) | GBS growth | GBS growth | GBS growth | GBS growth | GBS growth | No bacteria growth | No bacteria growth |

1. Our experiments showed that, without gentamicin washing mixed bacteria, GBS growth is almost completely inhibited and difficult to be separated, due to *Escherichia coli, Staphylococcus aureus* grows faster than the growth rate of GBS. In contrast, the mixed bacteria washed with normal saline containing gentamicin were cultured for 14 - 20 hours, cultured on Colombian blood plates for 18 -24 hours. We found the GBS were isolated easily in the 2nd, 3rd and 4^th^ areas of blood plates.

In summary, our method inhibited the growth of non-GBS other bacteria and allowed to make GBS to grow. Experiments showed that the method is an economic and good method.
